# China shares fossil treasures with the world

**DOI:** 10.1002/ar.25696

**Published:** 2025-05-26

**Authors:** Peter Dodson

**Affiliations:** ^1^ Department of Biomedical Sciences, School of Veterinary Medicine University of Pennsylvania Philadelphia Pennsylvania USA; ^2^ Department of Earth and Environmental Science University of Pennslyvania Philadelphia Pennsylvania USA

**Keywords:** China, dinosaurs, fossils, mammals, vertebrates

## Abstract

China has been a rich source of fossils for nearly a century, beginning with the discovery of so‐called Peking man (*Sinanthropus pekinensis*), known today as *Homo erectus pekinensis* in the mid 1920s. The first Chinese dinosaurs were described in 1929, the sauropod *Helopus* (now *Euhelopus*) and the ornithopod *Tanius,* described by the Swedish paleontologist Carl Wiman. Over the next six decades, further dinosaurs were described by Yang Zhongjian (C.C. Young) and his students Dong Zhi‐Ming and Zhao Xijin, but remained poorly known in the West. A golden age of Chinese paleontology began as spectacular feathered dinosaurs were described from Lagerstätten in northeastern China beginning in 1996. Today, China has more genera of dinosaurs than any country on earth. In addition to dinosaurs and birds, China has among the oldest fossil vertebrates on earth with Cambrian fish such as *Haikouella* and *Myllokunmingia,* one of the first fossil flowers with Early Cretaceous *Archaefructus,* and a rich fauna of mammals, including Early Eocene *Archicebus,* one of the earliest known fossil primates. Fossil mammals range from a Jurassic beaver‐tailed aquatic docodont, *Castorocauda,* to a Cretaceous gobiconodontid, *Repenomamus,* which had the nerve to munch on a baby dinosaur, to Ice Age elephants, woolly rhinoceros, horses, and saber‐toothed cats. Surprising new fossils of all kinds will continue to be discovered in China for decades to come.

When I was a graduate student more than half a century ago, I was enthralled by the Polish—Mongolian Paleontological Expeditions of 1967–1971 (Kielan‐Jaworowska & Barsbold, 1972), whose results began to appear just as I was cutting my teeth in paleontology. China did not register in my consciousness in the early days of my training. Mongolia yes, China no. However, in the later 1980s I became aware of China because of the ambitious China—Canada Dinosaur Project, led by my friends Dale Russell of the Canadian Museum of Nature and Phil Currie of the Tyrrell Museum of Palaeontology (subsequent Royal Tyrrell Museum of Palaeontology). This highly successful project resulted in tons and tons of fossils from Inner Mongolia and Xinjiang (Dong, 1993). I had the pleasure of meeting Professor Dong Zhi‐Ming on a visit to Ottawa in 1987. Dong, who died October 20, 2024, became a major inspiration in my career, and I dedicate this essay to his memory (Figure 1). In the early 1990s, China was the center of international attention because of highly publicized discoveries of huge numbers of beautifully preserved dinosaur egg clutches in Henan Province and adjacent areas of southeastern China. 1996 was a pivotal year for dinosaur discoveries in China. In August 1996, a Chinese farmer from Liaoning Province northeast of Beijing split a rock and was rewarded by a stunning discovery—a complete specimen of a small theropod dinosaur showing short bristle‐like feathers running along the midline of the back from the head to the tip of the tail. The plucky farmer sold one slab to the National Geological Museum in Beijing and the counterpart slab to the Nanjing Geological Institute, nearly 600 miles south of Beijing. The significance of the specimen was swiftly recognized by Professor Ji Qiang and was rushed into print in *Chinese Geology* under the name of *Sinosauropteryx*, meaning Chinese reptile wing (Ji & Ji, 1996). Photos of the fossil were shown to small groups of amazed attendees at the October 1996 annual meeting of the Society of Vertebrate Paleontology meeting at the American Museum of Natural History in New York. The cat was now out of the bag—China is a certified source of some of the most extraordinary fossils ever seen—the equal of the *Archaeopteryx* fossils from Bavaria in terms of exquisite preservation of soft tissues such as feathers and keratinous claw sheaths. Whereas the fossil record has been extremely frugal in Bavaria, feathered fossils of both non‐volant theropods and volant birds have been found in abundance not only in Liaoning, but elsewhere in China, including Gansu in northwestern China (You et al., 2006).

Prior to this stunning discovery, knowledge of Chinese dinosaurs and other fossils was not widely appreciated. Now the secret was out and China was a hot ticket for paleontologists. I made my first trip to China in 1995 to attend Sixth Symposium on Mesozoic Continental Ecosystems in Beijing a year prior to the electrifying discovery of *Sinosauropteryx*. This meeting changed the arc of my professional career. There I received the warm hospitality of Dong Zhi‐Ming, and met his young protégé, You Hailu (Figures 2, 3). Hailu had just finished his master's degree at the IVPP, and was charged with the task of shepherding foreign visitors on field excursions related to the meeting. His personality, facility with English, and general level of education and intelligence made him ideal for herding western scientists on unfamiliar territory. What really changed my life was inviting Hailu to come to Philadelphia to study with me at the University of Pennsylvania for his Ph.D., which he did. Inspired by Professor Dong, Hailu explored fossil beds in the remote Mazongshan region of northwestern Gansu Province not far from the border with the Peoples Republic of Mongolia. Two of the dinosaurs he described in his dissertation were the sauropod *Gobititan* (You, Tang, et al., 2003) and the hadrosauroid *Equijubus* (You, Luo, et al., 2003; You, Tang, et al., 2003).

My first research trip to China was in 1997, when my two students, Matt Lamanna and Josh Smith, joined Hailu for an expedition to fossil quarries in the now‐famous Early Cretaceous (Aptian) Yixian Formation in Liaoning northeast of Beijing. We reveled in splitting open slabs of the finely laminated volcanoclastic sediments filled with exquisitely preserved fossils that constitute the Jehol Biota (Smith et al., 2001). These beds constitute a classic Lagerstätte, the term applied to beds that preserve abundant and diverse fossils with soft parts of the sort rarely seen otherwise (Kimmig & Schiffbauer, 2024). Fossil fishes (*Lycoptera* sp.) are abundant, as are conchostracans (aquatic arthropod—*Eosestheria* sp.) and insect larvae (*Ephemeropsis*). We of course were not so fortunate as to discover any dinosaurs or feathered bird specimens, but these are important components of the rich and diverse Jehol Biota, as are salamanders, lizards, turtles, pterosaurs, mammals, and plants, including one of the earliest flower‐bearing plants, *Archaefructus* (Sun et al., 2002). Feathered birds, especially *Confuciusornis*, have been found in large numbers. Small feathered dinosaurs such as *Sinosauropteryx* and *Microraptor* are quite rare but exquisite. Specimens of *Psittacosaurus* also form part of the Jehol Biota, but fail to evoke the awe that the small theropods do.

Except for a study of *Archaeoceratops* (You & Dodson, 2003) I initially was not directly involved in Chinese dinosaur research, being distracted by projects in Montana and Egypt. Hailu completed his Ph.D. in 2002, and then returned to China. Soon he and his Chinese mentor, Professor Dong, named a ceratopsian fossil from Inner Mongolia *Magnirostris dodsoni* (“Dodson's big nose?”) in 2003 (You & Dong, 2003). As pleased as I was with this unexpected honor, I was even more pleased when Hailu invited me to return to China the following summer to engage in collaborative research with him and my students. I returned to China every year, accompanied by my students, for the following decade. Hailu brought me to Gansu Province in the northwest in 2004, where I met Dr. Li Daqing, who became a highly valued friend and colleague (Figure 4). Daqing was trained as a stratigrapher and invertebrate paleontologist, but he turned his hand to dinosaurs and explored a region where dinosaurs were not yet well known. Northwestern Gansu adjacent to Mongolia (and immediately west of the Chinese province of Inner Mongolia) was first visited in 1930 by the Sino‐Swedish Expedition led by Sven Hedin. Fragmentary remains of several different kinds of dinosaur were described by Bohlin in 1953, but none were taxonomically diagnostic. In 1988 the China—Canada Dinosaur Project made a cursory visit to this region but did not locate sufficient fossils to make focus there rewarding, and so continued on to Xinjiang, where great discoveries were made. Professor Dong, however, realizing the potential of the Mazongshan region of Gansu, organized the Sino‐Japanese Silk Road Dinosaur Expeditions of 1992 and 1994, and recovered high quality dinosaurian fossils, including the basal ceratopsian *Archaeoceratops* (Dong & Azuma, 1997; You & Dodson, 2003).

In the meantime, Li Daqing, who was based not in Beijing but in Lanzhou, the capital of Gansu, chose to devote his efforts to collecting in the Mazongshan region of Gansu. He collected with great distinction and amassed a museum filled with fossil treasures. When I first visited Daqing's lab in 2004, accompanied by Hailu, my eye quickly fell on a three‐dimensionally preserved fossil skull that I instantly recognized as (1) a basal ceratopsian, and (2) absolutely new to science. I was working from a position of strength, having earlier written the definitive book on horned dinosaurs (Dodson, 1996). Daqing invited me to study and describe the specimen, which I did with the greatest pleasure. We published the description a year later, and chose to name it in honor of my wife, Dawn: hence, *Auroraceratops* (You et al., 2005). The honoree was pleased, even if the dinosaur is old and, frankly, a bit ugly. There is a back story to the name, and that is that I unwisely chose to name my first dinosaur, *Avaceratops* (Dodson, 1986), in honor of Ava Cole, co‐discoverer of that fossil. It took me 19 years to realize and overcome that marital *faux pas*. In any case, follow up field work with Daqing's crew of dedicated workers and my students and I turned up several scores of specimens of *Auroraceratops* of varying sizes (Figures 5, 6). These specimens formed the core of my student Eric Morschhauser's (2012) dissertation and subsequent monograph (You et al., 2018; Morschhauser et al., 2018). From 2005 to 2024, a total of 14 genera of dinosaurs have been described from Gansu, all of them collected by Li and his superb team. Hailu has been author or co‐author of 12 of these, making me a very proud *Doktorvater*.

Paleontology has been humming at a fever pitch in China for several decades. It was not always so. The history of paleontology in China dates back to the 1920s. The 1920s were a turbulent time in Chinese history, and the People's Republic of China did not yet exist. The era of dinosaur paleontology in China dawned more quietly than in Mongolia, which was explored by the highly publicized American Museum of Natural History Expeditions of 1922 to 1930 led by the flamboyant Roy Chapman Andrews. The first Chinese dinosaurs that are still recognized as valid include a sauropod excavated in Shandong Province in 1923 by the Austrian paleontologist Otto Zdansky and Chinese geologist Tan Xi‐Chou, and a hadrosaur from the same region. The skeletons were shipped to Sweden, where they were described as *Helopus* (now *Euhelopus*) and *Tanius*, respectively, by Carl Wiman in 1929. Fortunately, Chinese science was not far behind, in the person of C.C. Young (now known by his Chinese name of Yang Zhongjian, 1897–1979). Educated in Germany, Yang described his first dinosaurs in 1932, several species of *Psittacosaurus*, and thereafter conducted excavations in Yunnan, Sichuan, Shandong, Xinjiang, and Gansu. He was the founder and first director of the Institute of Vertebrate Paleontology and Paleoanthropology (IVPP) in Beijing, well known to paleontologists around the world. His legacy includes such important dinosaurs as *Lufengosaurus, Yunnanosaurus, Mamenchisaurus, Omeisaurus* and *Tsintaosaurus*. He trained many paleontologists, most importantly Zhao Xijin (Chao Shichin) and Dong Zhi‐Ming, who kept Young's legacy of dinosaur paleontology alive after Young's death in 1979. Dong has collected and described many dinosaurs during his career, and ranks among the most prolific describers in the history of dinosaur paleontology; his only peers are O.C. Marsh and Jose Bonaparte, and also Xu Xing. Among Dong's 30 genera of dinosaurs are *Archaeoceratops, Huayangosaurus, Shunosaurus*, and *Tuojiangosaurus*. Dong also described several genera of marine reptiles and pterosaurs. Dong and Zhao published primarily in Chinese journals, and so their discoveries were not well known in the West during the Cold War.

**FIGURE 1:**
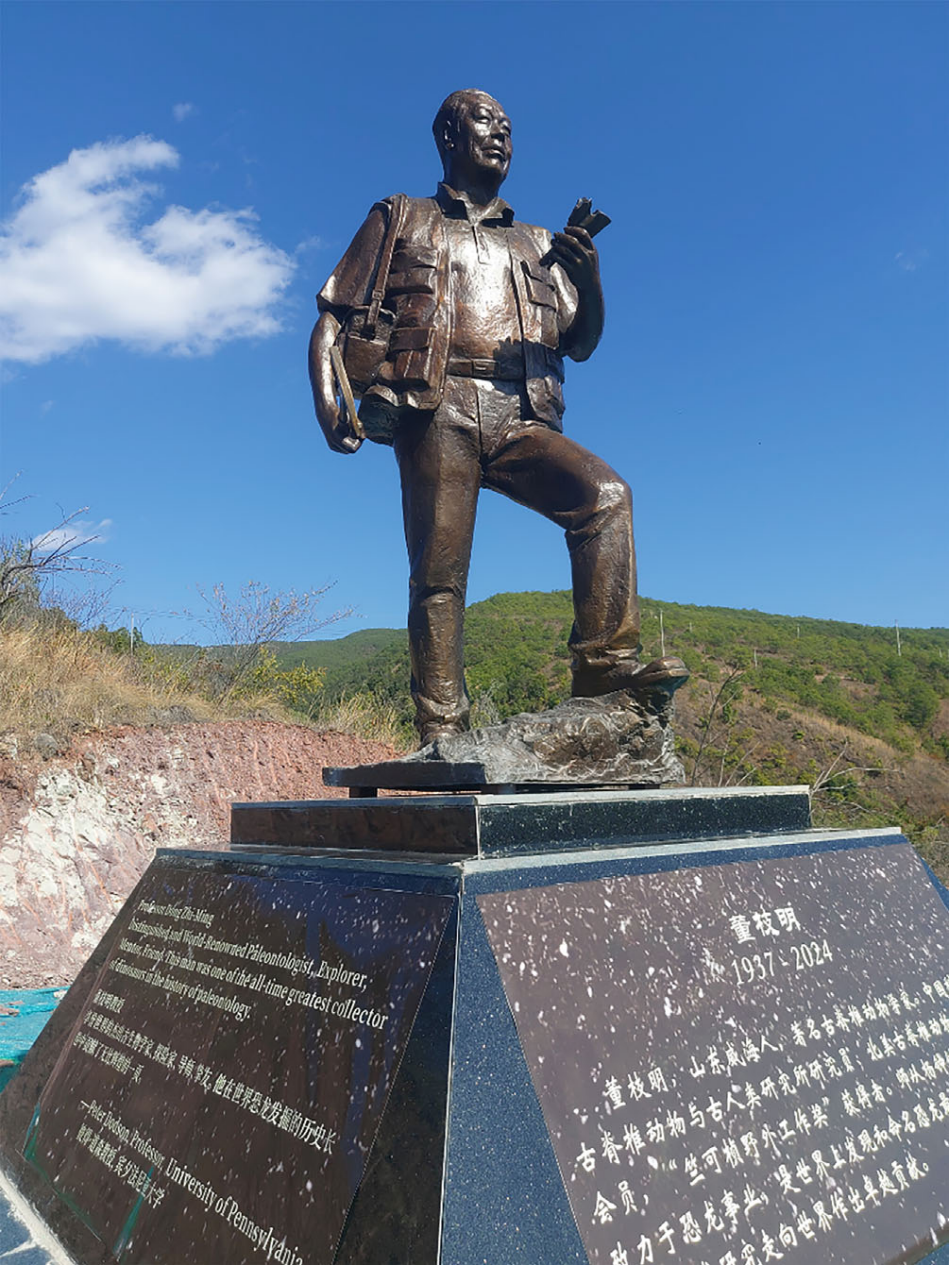
Statue in honor of Dong Zhi‐Ming (1937–2024) erected January 2025 at Lufeng Dinosaur Valley, a dinosaur park founded by Dong at a fossil site in Yunnan Province. The English inscription, written by the author, reads “Professor Dong Zhi‐Ming distinguished and World‐Renowned Paleontologist, Explorer, Mentor, Friend. This man was one of the all‐time greatest discoverers of dinosaurs in the history of paleontology”. Photo courtesy of You Hailu.

**FIGURE 2:**
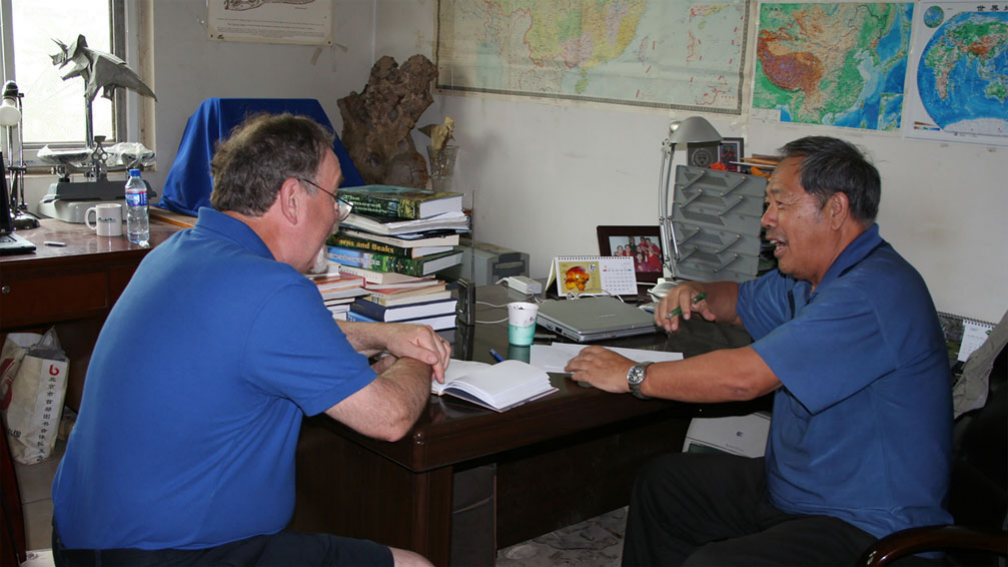
Dodson discusses Chinese dinosaurs with Dong Zhi‐Ming in You Hailu's office in Beijing, summer 2007.

**FIGURE 3:**
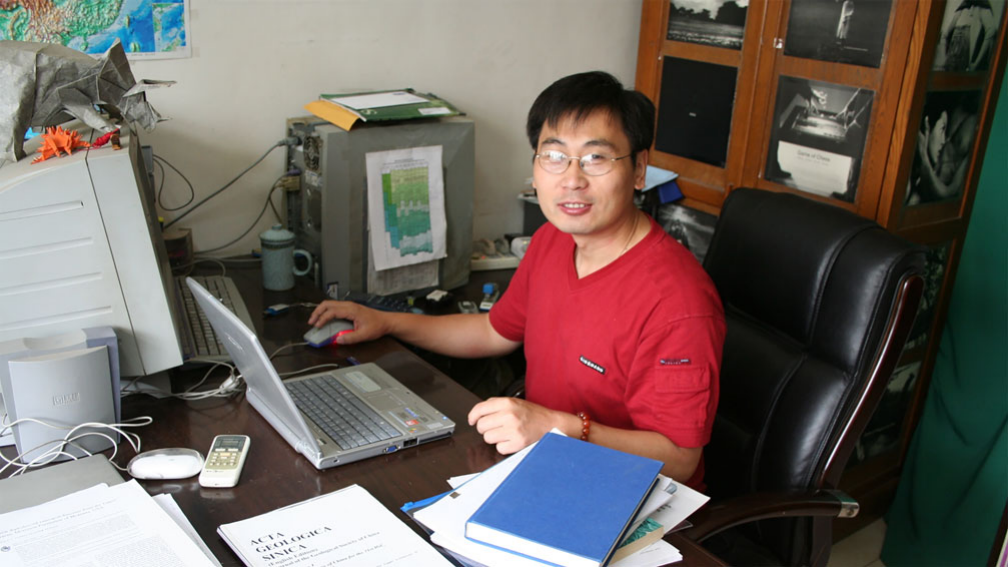
Dr. You Hailu (Ph.D. UPenn, 2002) in his office in Beijing.

**FIGURE 4:**
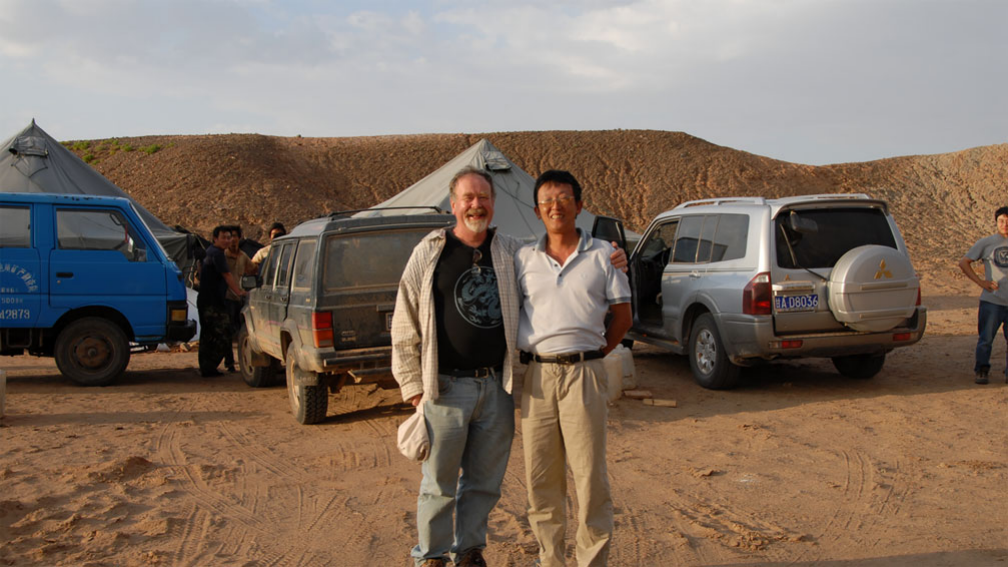
Dodson and Dr. Li Daqing in camp, Yujingzi Basin, Mazongshan Region, Gansu Province.

**FIGURE 5:**
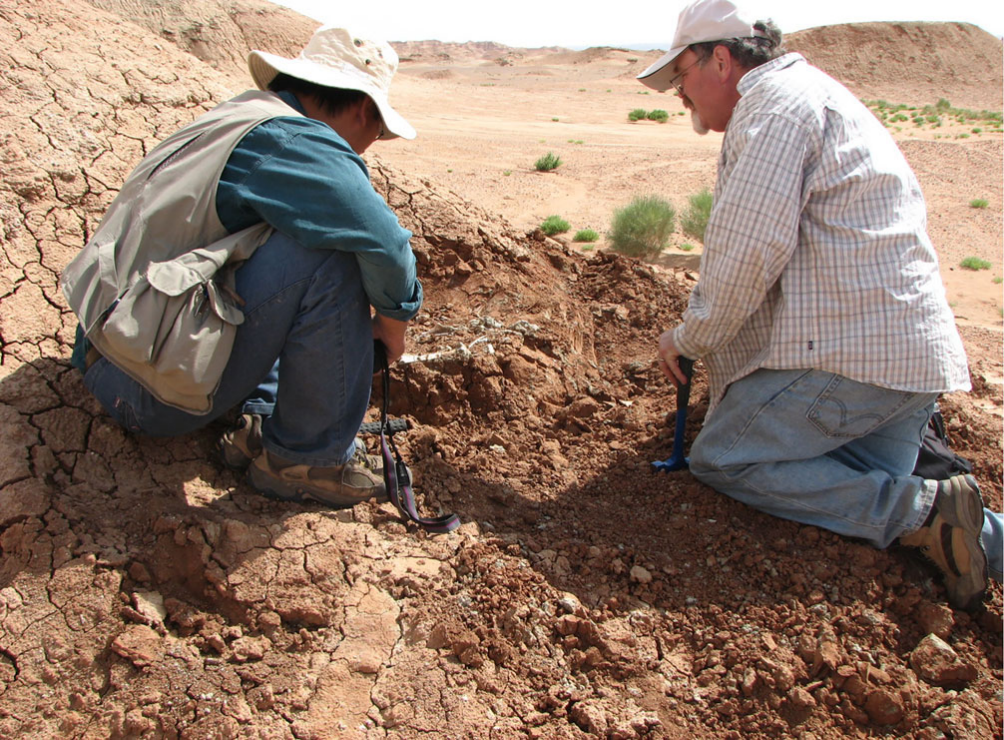
You and Dodson examine a specimen of *Auroraceratops* in the field, Yujingzi Basin.

**FIGURE 6:**
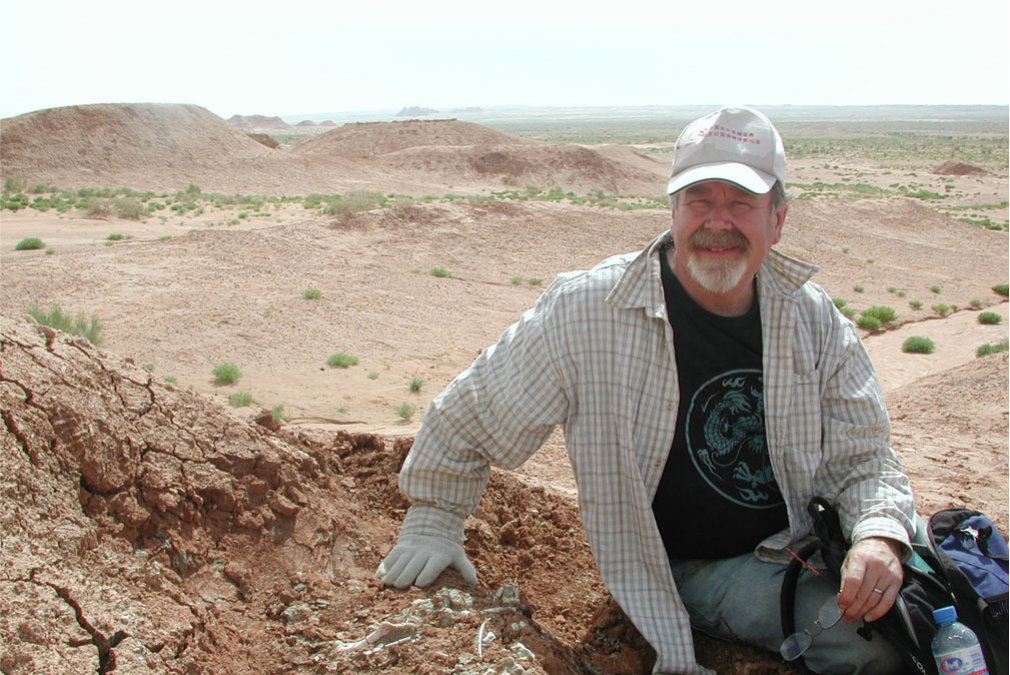
Dodson examines a specimen of *Auroraceratops* in the field, Yujingzi Basin. Photo courtesy of You Hailu.

**FIGURE 7:**
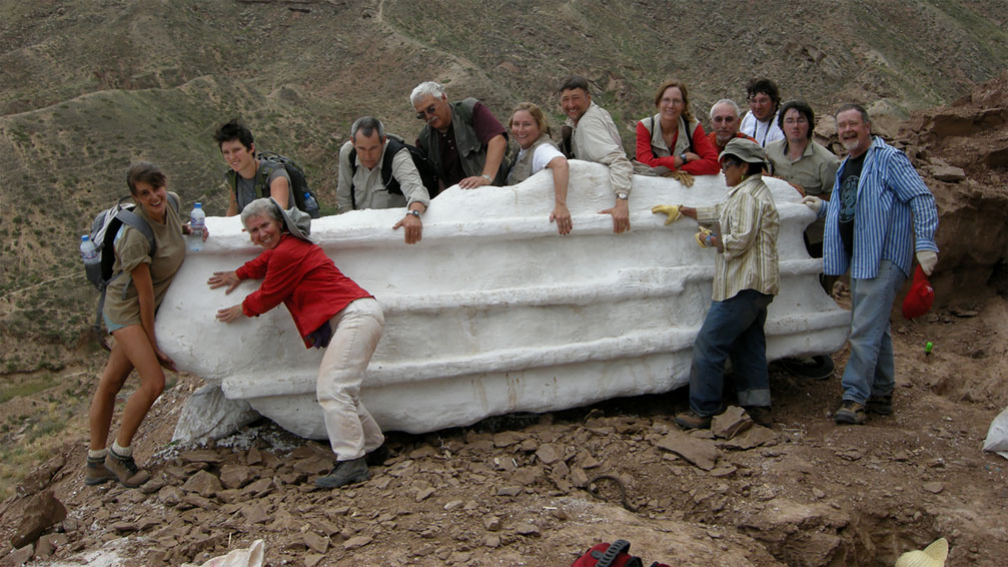
Paleo tourists led by Dr. You Hailu pose with an enormous block containing cervical vertebrae of the very large sauropod *Daxiatitan*. The specimen was collected by Dr. Li Daqing and his team. It was successfully pulled downslope by his crew without the aid of machines. Yongjing County, Lanzhou‐Minhe Basin, Gansu, 2008. Penn undergraduate Jessie Atterholt is at the left, Dodson at the extreme right. Photo courtesy of You Hailu.

**FIGURE 8:**
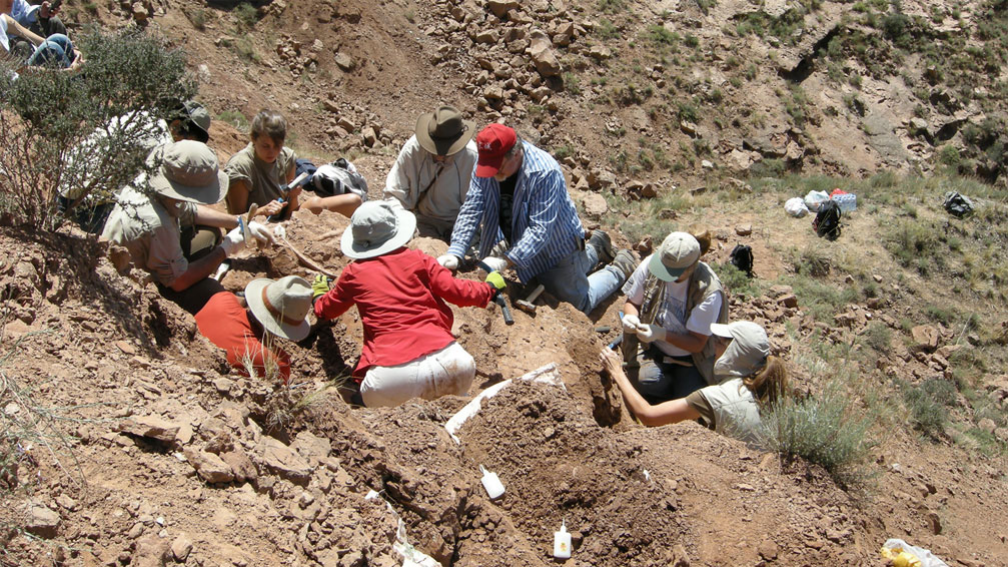
Paleo tourists led by Dr. You Hailu engage in significant excavation. The specimen collected turned out to be the type specimen of the polacanthine ankylosaur, *Taohelong*. Dodson right, Atterholt center rear. Yongjing County, Lanzhou–Minhe Basin, Gansu, 2008. Photo courtesy of You Hailu.

**FIGURE 9:**
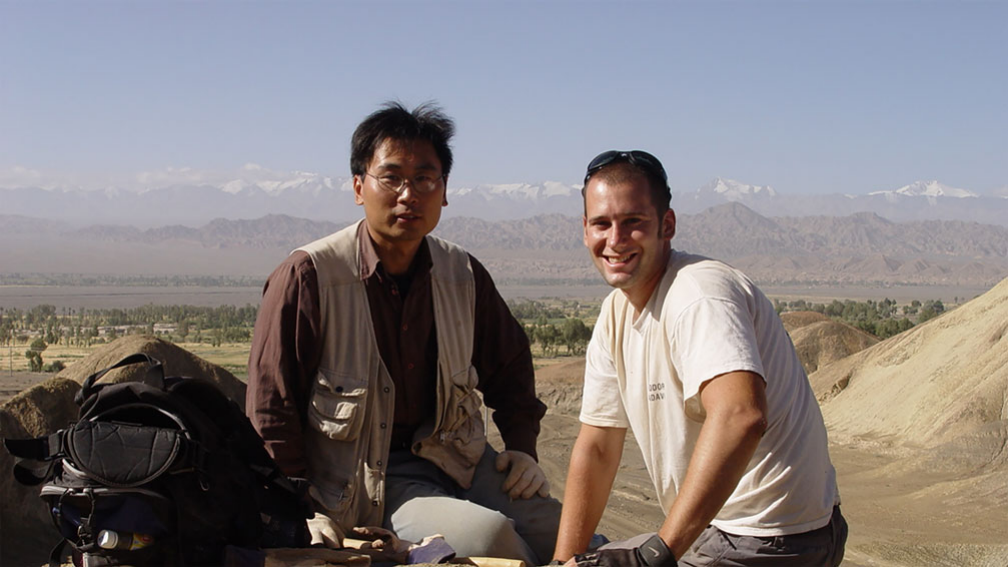
You Hailu and Matt Lamanna discover a fossil bird at Changma, Yumen City, Gansu, 2004. Photo courtesy of You Hailu.

Following the description of *Sinosauropteryx* in 1996, Chinese paleontologists reached out to the world, regularly attending scientific conferences in the West, and many highly productive collaborations developed with researchers in the UK (Museum of Natural History, University of Bristol), Canada (Royal Tyrrell Museum of Paleontology, University of Alberta, Canadian Museum of Nature), and the United States (American Museum of Natural History, University of Pennsylvania, George Washington University, University of Chicago), to mention but a few. Xu Xing of the IVPP has now discovered and described more dinosaurs than any other paleontologist in history. Xu's list of species exceeds 60, and he has lost count! Many Chinese dinosaurs are now described in Open Access English language international journals, and are among the best known dinosaurs in the world, especially the spectacular feathered variety. In fact, the latest of the latter is *Baminornis* from Fujian Province, published in February 2025 (Chen et al., 2025). This is a long‐awaited find of a precocious Jurassic bird that lacked the long bony tail of *Archaeopteryx*. For many years, beginning with the great dinosaur rush of the 1870s, the United States has had more kinds of dinosaurs than any other country (Dodson, 1990). China was number three on the list after Mongolia. I naively thought that the U.S. would remain number one forever more, but in 2007 China surpassed the United States in the number of dinosaur genera (Dodson, 2009), and the gap has widened as the years have gone by. When I counted in 2023, I tallied China at 279 genera and the United States at 174 (with Argentina closing fast at 155 genera). You Hailu (pers. comm., 2025) has compiled some very interesting figures about the dinosaurs of China. Liaoning, the site of the electrifying discoveries of 1996, boasts the richest, most diverse dinosaur fauna by far in all of China, with 62 taxa reported so far. Inner Mongolia comes in a distant second with 41 genera. This is followed by Sichuan (38) and Xinjiang (30). Yunnan (25) is the site of ongoing exploration, and further discoveries will be reported in the near future. Not only does China possess dinosaurs in abundance, but the often high quality of preservation may reveal remarkable details. We have come to accept the concept of feathered theropods, so beautifully documented in China (e.g., Norell & Xu, 2005), cementing the phylogenetic link between theropods and birds. What is truly surprising is to find filamentous appendages in ornithischians unrelated to theropods or birds. One such dinosaur is *Tianyulong*, a very basal ornithischian from Early Cretaceous deposits in Liaoning (Zheng et al., 2009). Another example is seen in a more derived ornithischian, *Psittacosaurus*, a bipedal small‐bodied basal ceratopsian. One spectacular specimen from Liaoning (Mayr et al., 2002) shows a luxuriant series of 16 cm long keratinous bristles adorning the base of the tail. The spines project caudodorsally and suggest the appearance of a wayward porcupine.

I write this essay as if dinosaurs were the only fossils in China. Of course, nothing could be farther from the truth, as the present volume attests. China has a rich record of fossil mammals and near‐mammals, both Mesozoic and Cenozoic, fishes of every sort, amphibians, non‐dinosaurian reptiles including Mesozoic marine reptiles and birds, not to mention insects and plants. An extremely consequential discovery was made in limestone karst deposits of Middle Pleistocene age at Zhoukoudian, only 50 km southwest of Beijing. Here during the 1920s an international scientific party of Chinese, Swedish, Austrian, French and Canadian paleontologists discovered a rich Ice Age fauna of mammals, including multiple specimens of “Peking man,” originally named *Sinanthropus pekinensis* (“Chinese ape from Peking”) but now known as *Homo erectus pekinensis*. Other faunal elements include giant deer, straight‐tusked elephant, woolly rhinoceros, horses of the genus *Equus*, diverse carnivores including wolves and saber‐toothed cats, as well as rodents. The story of discovery, description and disposition of the Zhoukoudian fossils is a fascinating one that played out against the tapestry of tumultuous world events that culminated in World War II (Aczel, 2007; Boaz & Ciochon, 2004). China boasts one of the oldest fossil primates known to science, Early Eocene *Archicebus achilles* from Hubei Province (Ni et al., 2013). Mesozoic mammals include the Middle Jurassic swimming beaver‐tailed docodont, *Castorocauda lutrasimilis* (Ji et al., 2006). A recently‐described Jurassic gliding near‐mammal, a euharamiyid, is so well preserved that the melanosomes of its pelage show that is was dark‐colored, consistent with nocturnal habits (Li et al., 2025). A remarkable Early Cretaceous triconodont mammal is *Repenomamus giganticus* whose stomach contents include remains of hatchling *Psittacosaurus* dinosaurs (Hu et al., 2005). The earliest vertebrates of all come from the Early Cambrian Chengjiang Lagerstätte of Yunnan Province of southwestern China (Hou & Bergström, 2003). The soft‐bodied creatures of this marine deposit are 10 million years older than the famous Middle Cambrian Burgess Shale of British Columbia. Two primitive agnathan fishes from the Chenjiang Biota are *Haikouella* and *Myllokunmingia* (Shu et al., 1999), humble precursors of the great radiation of vertebrates on land and sea the developed over the next 520 million years.

The present volume ably represents some of the great diversity of fossils, including not merely vertebrates but insects from the Triassic and Jurassic, and brachiopods from the Permian. We learn of jawless fishes from the Devonian and an aquatic archosauromorph from the Triassic of Yunnan. We are treated to reports of the shoulder girdle and tooth structure in a pterosaur from the Cretaceous of Xinjiang, and a description of an enantiornithine bird from the Cretaceous of Liaoning. Our dinosaur is a hadrosaur from the historical Shandong, which provided *Tanius* and *Euhelopus* in 1929. A protomammal is *Bienotheroides* from the Jurassic of Xinjiang. Reports of fossil mammals range from the Eocene to the Pleistocene. There is something for everyone in the fossil record of China.

In summary, China teams with interesting fossils from the mountain heights of Tibet and the desert wastes of Xinjiang in the west to the salty shores of Liaoning and Shandong in the east, and from the steppes of Inner Mongolia in the north to mountainous Yunnan in the southwest. Surprising discoveries of all kinds will continue to amaze and delight with no end in sight.

China has been very good to me. It has been the greatest privilege of my life to have trained a Chinese student, You Hailu, who is now a senior scientist at the prestigious Institute of Vertebrate Paleontology and Paleoanthropology (IVPP) in Beijing, which may be compared to the Museum of Natural History of the Smithsonian Institution in Washington, DC. Hailu's mentor, Dong Zhiming, became my friend, as did Li Daqing, Hailu's colleague in Gansu Province. I enjoyed the hospitality of Xu Xing at the IVPP while Hailu was a graduate student at the University of Pennsylvania, my home institution, and also of Ji Qiang at the Institute of Geology in Beijing. In addition to You Hailu, six other Ph.D. students of mine worked in China: Kyo Tanoue, Eric Morschhauser, Matt Lamanna (Figures 7, 8, 9), Josh Smith, Brandon Hedrick and Liguo Li (Dodson, 2020). A number of undergraduates also accompanied me to China. With my Chinese colleagues and my students, we described and named three genera and four species of dinosaurs. Furthermore, You Hailu has his own students who are diligently contributing to the fossil record of China, with concentrations in Gansu and Yunnan. A century ago paleontology began in China with the help of international scientists. Today paleontology in China is the envy of the world.

## AUTHOR CONTRIBUTIONS

**Peter Dodson:** Conceptualization; writing – original draft; writing – review and editing.
